# Optimization of DNA Extraction for RAPD and ISSR Analysis of *Arbutus unedo* L. Leaves

**DOI:** 10.3390/ijms12064156

**Published:** 2011-06-22

**Authors:** Olga Sá, José Alberto Pereira, Paula Baptista

**Affiliations:** Mountain Research Centre, School of Agriculture, Polytechnic Institute of Bragança, Campus St^a^ Apolónia, Apartado 1172, 5301-855, Bragança, Portugal; E-Mails: olga_de_sa@hotmail.com (O.S.); jpereira@ipb.pt (J.A.P.)

**Keywords:** *Arbutus unedo* L., strawberry tree, DNA isolation, RAPD, ISSR

## Abstract

Genetic analysis of plants relies on high yields of pure DNA. For the strawberry tree (*Arbutus unedo*) this represents a great challenge since leaves can accumulate large amounts of polysaccharides, polyphenols and secondary metabolites, which co-purify with DNA. For this specie, standard protocols do not produce efficient yields of high-quality amplifiable DNA. Here, we present for the first time an improved leaf-tissue protocol, based on the standard cetyl trimethyl ammonium bromide protocol, which yields large amounts of high-quality amplifiable DNA. Key steps in the optimized protocol are the addition of antioxidant compounds—namely polyvinyl pyrrolidone (PVP), 1,4-dithiothreitol (DTT) and 2-mercaptoethanol, in the extraction buffer; the increasing of CTAB (3%, w/v) and sodium chloride (2M) concentration; and an extraction with organic solvents (phenol and chloroform) with the incubation of samples on ice. Increasing the temperature for cell lyses to 70 °C also improved both DNA quality and yield. The yield of DNA extracted was 200.0 ± 78.0 μg/μL and the purity, evaluated by the ratio A_260_/A_280_, was 1.80 ± 0.021, indicative of minimal levels of contaminating metabolites. The quality of the DNA isolated was confirmed by random amplification polymorphism DNA and by inter-simple sequence repeat amplification, proving that the DNA can be amplified via PCR.

## 1. Introduction

The strawberry tree, *Arbutus unedo* L., is a typical evergreen plant of the Mediterranean basin, as well as of other regions with hot summers and mild rainy winters. It is native to Greece, Lebanon, Southern Europe and Anatolia [1]. In Portugal, this specie appears mainly in the south (Algarve region), although it can be found in sparse distribution throughout the country [2]. The strawberry tree plays an important role in the economy of the regions where it occurs. The production of alcoholic drinks from its fruits, such as liqueurs, and especially brandies, represents the main income for farmers [3]. More recent uses are related with biomass production and floriculture [4]. The strawberry tree has also social and cultural importance since fruits continue to be used in regional gastronomy. They could be applied in the preparation of jams, jellies and marmalades, and in the confectionary of pies and pastry fillings [3]. Both fruits and leaves are also used in folk medicine to treat several diseases due to their recognized phytopharmaceutical properties [5–8]. Additionally, this specie has landscape importance especially due to its attractive red fruits in the fall and winter, and pinkish-white flowers in the fall.

During the last years several occurrences have caused a decline of strawberry tree in Portugal. Since this phenomenon may put the specie in danger it is extremely urgent to adopt management and conservation strategies. It is therefore essential to characterize, morphologically and genetically, the different populations of *A. unedo*, which are considered the keys elements in management programs.

However, to the author’s knowledge, the intraspecific genetic biodiversity is not known. This could be related to the difficulty to isolate high-quality DNA from *A. unedo* tissues, a key element in such studies that use various molecular techniques. The difficulties encountered while working with this specie were caused by the presence of high amounts of polyphenols, polysaccharides, tannins and other secondary metabolites [9,10]. In addition, these contaminants interfere in downstream reactions such as DNA restriction, amplification and cloning [9].

Several protocols for DNA extraction have been successfully applied to plant species [9,11,12], which were further modified to extract high-quality DNA from plants containing such contaminants [13–16]. However, our research group previously tested these protocols as well as other unreported methods, and none of them proved to be suitable for extracting DNA from *A. unedo* leaves. All the protocols described low yields, degraded and impure DNA that could not be amplified in the polymerase chain reaction (PCR).

Thus, the present study aims to improve Doyle and Doyle method [11], by modifying some aspects of the procedures and extraction buffer composition, with an attempt to isolate high-quality DNA from *A. unedo* leaves. Random amplified polymorphic DNA (RAPD’s) reactions and inter-simple sequence repeat (ISSR) amplification were also performed in order to evaluate the suitability of the extracted DNA for PCR-based techniques. As far as we know, this is the first report on DNA extraction from *A. unedo*, and we expect that this optimized protocol can be an incentive to perform studies in order to investigate the genetic diversity among this specie.

## 2. Results and Discussion

The strawberry tree is a typical plant specie of the Mediterranean basin. This specie plays diverse roles both in the natural environment and as a resource in rural areas, with recognized traditional uses in the food industry, phytochemistry, medicine and ornamental plant production. Therefore, it is urgent to preserve the genetic resources of *A. unedo*, integrating the conservation issue with their sustainable exploitation. This can only be achieved once the existent genetic diversity of *A. unedo* is clear, which is practically unknown. Various types of DNA-based molecular techniques are used to evaluate the genetic variability in plants. These approaches require both high-quality and quantity DNA, for which *A. unedo* presents a great challenge.

**Figure 1:**
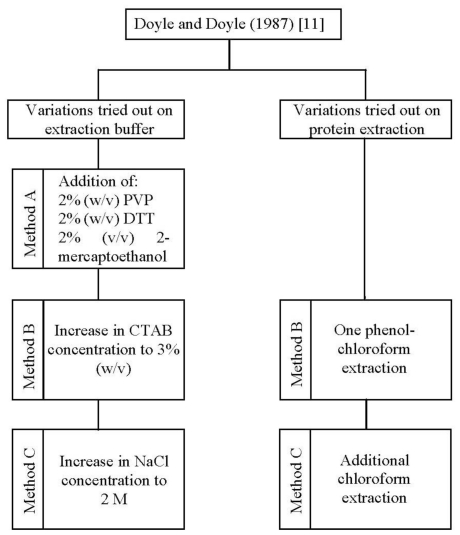
Variations tried for the optimization of DNA extraction from *Arbutus unedo* leaves.

In the present study, one standard [11] and three improved methods (method A, B, and C) for DNA isolation were applied to *A. unedo* leaves (Figure 1).

Firstly, we tested the CTAB method reported by Doyle and Doyle [11], which proved to be inadequate. With this method no DNA was extracted (Table 1). This is probably due to the specific characteristics of this plant, like the presence of polyphenols, tannins, polysaccharides, proteins and other secondary metabolites [10,17,18], which either lead to embedding of DNA into a sticky gelatinous matrix [19] or promote DNA degradation [20]. Taking into consideration the traditional application form of *A. unedo* leaves, the compounds that provide the therapeutic efficacy to the plant could be also a problem in the isolation procedure by binding with the DNA and precipitating along with it [21]. Accordingly, we have modified Doyle and Doyle–CTAB [11] protocol to improve DNA yield and quality.

**Figure 2:**
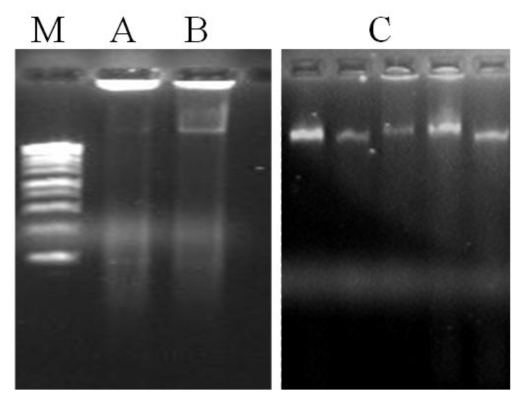
Electrophoretic pattern of DNA extracted by the different modified CTAB methods (method A, B, and C) from *Arbutus unedo* leaves. M–1 Kb molecular weight marker (Thermo Scientific, Loures, Portugal). The electrophoresis was performed in 1.2% (w/v) agarose gel.

The first yield improvement was achieved by adding antioxidant compounds to the extraction buffer (method A). The addition of PVP, DTT and 2-mercaptoethanol allowed an increase in the DNA yield from 0 to 85.4 μg/μL (Table 1). This procedure proved to be crucial to reduce DNA degradation by oxidized polyphenols formed during cell lyses. In fact, it is known that *A. unedo* leaves are rich in polyphenols like flavonoids [10,17,18], which have been associated with the degradation of genomic DNA [22]. PVP act as an adsorbent of polyphenols [20] while DTT and 2-mercaptoethanol inhibit the oxidation of the same compounds. Although DNA yield was incremented in method A, another problem persists by the presence of contamination compounds in the DNA samples, particularly proteins and polysaccharides, as visualized in the agarose gel (Figure 2) and confirmed by the low A_260_/A_280_ ratio obtained (1.48, Table 1). Complete removal of polysaccharides during DNA isolation assumes critical importance due to their well-established interference problems, namely failure of DNA amplifications during PCR due to inhibition of *Taq* polymerase activity [23].

Thus, a further optimization was obtained by increasing the concentration of CTAB (method B) and in addition, that of NaCl (method C) in the extraction buffer (Figure 1). The combination of a high concentration of CTAB (3%, w/v) and NaCl (2 M), as performed in method C, increased the genomic DNA yield by 2.34 and 1.80 fold in comparison to methods A and B, respectively (Table 1). This step proved to be very critical for the recovery of pure DNA in the entire isolation process. The use of a high concentration of NaCl has been previously pointed to be suitable for the removal of polysaccharides from DNA solutions by increasing their solubility in ethanol, and thus preventing its co-precipitation with DNA [24,25]. NaCl combination with the cationic detergent CTAB has also been proved to be beneficial in DNA isolation from polysaccharide-rich plants [26]. To exclude protein impurities we tested one phenol-chloroform extraction (method B) followed by an additional chloroform extraction (method C) (Figure 1). The results obtained showed that the use of organic solvents have substantially removed proteins. In fact, method C has shown to extract DNA with higher purity (A_260_/A_280_ ratio equal to 1.80) than method A (A_260_/A_280_ ratio equal to 1.48) where no organic solvent extraction has been performed (Table 1). In addition, the elimination of proteins was also favored by the incubation of samples on ice after organic solvent addition. This result suggested that using only DTT and 2-mercaptoethanol in the extraction buffer, as well as protein-hydrolyzing enzymes like proteinase K, are not sufficient to remove proteins. Thus, the combination of high concentration of CTAB (3%, w/v) and NaCl (2 M) in the extraction buffer along with one wash with phenol:chloroform:isoamyl alcohol, followed by another with chloroform:isoamyl alcohol proved to be very effective to extract sufficient quantities of high-quality DNA from *A. unedo* leaves. The agarose gel electrophoresis of total genomic DNA showed high molecular weight DNA, with no sign of degradation and contamination (Figure 2). It is also worth mentioning that increasing the temperature used for cell lyses to 70 °C was helpful to improve both DNA quality and yield. With the original incubation temperature (60 °C) the yield and quality of DNA extracted was lower (data not shown).

**Figure 3:**
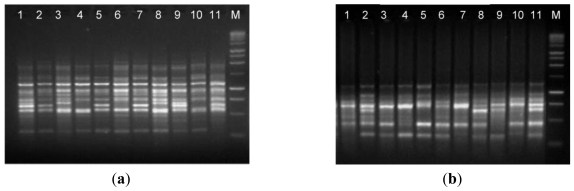
Amplification of DNA from 11 *Arbutus unedo* individuals (Lanes 1-11). (**a**) Random amplified polymorphic DNA (RAPD) using primer OPA-02; (**b**) and inter-simple sequence repeat (ISSR) amplification using the primer (CA)_8_A. M: 1 Kb molecular weight marker (Thermo Scientific, Loures, Portugal). The electrophoresis was performed in 2% (w/v) agarose gel.

The suitability of isolated DNA from the optimized protocol (method C) in molecular techniques was assessed by RAPD and ISSR analyses, which are useful to evaluate the genetic diversity and phylogenetic relationship. The results obtained shows that the DNA extracted from the optimized protocol was of suitable quality to screen levels of genetic diversity using both RAPD and ISSR and proving that the DNA can be amplified via PCR (Figure 3). The RAPD and ISSR patterns showed considerable genetic variation between *A. unedo* individuals from different geographic origin.

## 3. Experimental Section

### 3.1. Plant Material

In February of 2009, fresh and healthy leaves of *A. unedo* were randomly sampled from 19 individuals of different geographically representative natural populations in the Trás-os-Montes region (Northeast of Portugal). After collection, the leaves were ground to a fine powder in a mortar with a pestle in the presence of liquid nitrogen, and stored at −80 °C until DNA extraction.

### 3.2. DNA Isolation

The commonly used DNA isolation method, developed by Doyle and Doyle [11], using cetyl trimethyl ammonium bromide (CTAB) in the extraction buffer was tried in the beginning. Since results proved to be unsatisfactory, we have developed and tested three modified CTAB protocols (method A, B and C). In these new protocols we optimized the composition of the extraction buffer and introduced an additional step for protein removal (Figure 1). CTAB extraction buffer composition was firstly modified by employing polyvinyl pyrrolidone (PVP), 1,4-Dithiothreitol (DTT) and 2-mercaptoethanol (method A), followed by an increase in CTAB (method B) and sodium chloride (method C) concentrations. To exclude protein impurities we tested one phenol-chloroform extraction (method B) followed by an additional chloroform extraction (method C). The optimized procedure, which allowed the great improvement on both DNA yield and purity, was described as follows.

#### 3.2.1. Reagents and Solutions

The extraction buffer consisted of 3% (w/v) CTAB (Sigma, Sintra, Portugal), 100 mM Tris-HCl pH 8.0 (CalBiochem, Lisbon, Portugal), 20 mM EDTA pH 8.0 (Merck, Lisbon, Portugal) and 2 M sodium chloride (NaCl; Merck, Lisbon, Portugal). After being autoclaved for 20 min, 2% (w/v) PVP (mol wt 40.000; Sigma, Sintra, Portugal), 2% (w/v) DTT (Sigma, Sintra, Portugal) and 2% (v/v) 2-mercaptoethanol (Merck, Lisbon, Portugal) were added to the extraction buffer, immediately before use. In addition, phenol: chloroform:isoamyl alcohol (25:24:1, v/v/v, from Fluka, Sintra, Portugal), chloroform:isoamyl alcohol (24:1, v/v, from Panreac, Cascais, Portugal), TE buffer (10 mM Tris-HCl pH 8.0, 1 mM EDTA), 70% (v/v) ethanol (Merck, Lisbon, Portugal), proteinase K (20 mg/mL, from Sigma, Sintra, Portugal) and ribonuclease A (10 mg/mL, RNase-A, from Sigma, Sintra, Portugal) were prepared. Absolute iso-propanol (Merck, Lisbon, Portugal) was also required.

#### 3.2.2. DNA isolation Protocol

Ground leaves tissues (approximately 100 mg) were transferred to a 2 mL micro tube containing 1.3 mL of pre-heated (70 °C) extraction buffer. The tube was mixed by inversion and left to stand for 2 min at room temperature. After that, 10 μL of proteinase K (20 mg/mL) was added and the mixture was mixed again by inversion for 1 min. The mixture was incubated at 70 °C in a water bath for 30 min with occasional mixing. The tube was centrifuged at 10,000 rpm, for 5 min at 4 °C and the supernatant was transferred to a clean 2 mL micro tube. An equal volume of phenol:chloroform:isoamyl alcohol (25:24:1) was added, mixed by using gentle inversion for 5 min, incubated on ice for 10 min and centrifuged at 10,000 rpm for 5 min at 4 °C. The supernatant was transferred to a clean 2 mL micro tube and an equal volume of chloroform: isoamyl alcohol (24:1) was added. The tube was then gently inverted for 5 min, incubated on ice for 10 min and centrifuged at 10,000 rpm for 5 min at 4 °C. The upper aqueous phase was transferred to a clean 1.5 mL micro tube and DNA was precipitated by adding one volume of ice-cold iso-propanol (−20 °C), mixed by gentle inversion until the homogeny phase appeared, incubated at −20 °C for 1 hour, and centrifuged at 13,000 rpm for 20 min at 4 °C. The obtained pellet was washed with 500 μL of ice-cold 70% ethanol and centrifuged again at 13,000 rpm for 5 min at 4 °C. The supernatant was discarded and the pellet was air-dry for 20 min at room temperature. Finally, the pellet was re-suspended in 50 μL of deionized water or TE buffer and stored at −20 °C. Sometimes DNA could be contaminated with RNA. In this case, it is necessary to perform an additional step, by adding 1 μL RNase-A (10 mg/mL) to the sample and to incubate it for 30 min at 37 °C.

### 3.3. Concentration, Purity and Quality of the DNA Extracted

The quantity and quality of the DNA obtained were assessed spectrophotometrically at 260 and 280 nm, and the A_260_/A_280_ ratio was used to assess contamination with proteins. This spectrophotometric analysis was performed in triplicate on the samples of extracted DNA, in a PG Instruments Ltd. T70 UV/VIS spectrometer. In order to verify DNA integrity, 3 μL DNA were subjected to gel electrophoresis on 1.2% (w/v) agarose gel, stained with ethidium bromide [27], visualized under a UV transilluminator and photographed using the Stratagene Eagle Eye II.

### 3.4. RAPD and ISSR Amplifications

RAPD and ISSR analysis were used to test the quality and performance of the DNA extracted from method C, which proved to be the most efficacious compared to others methods tested in the present study (see results).

RAPD reactions were performed in a volume of 25 μL containing 20 ng of template DNA, 10 × PCR Buffer (10 mM Tris HCl, pH 8.3; 50 mM KCl), 2 mM MgCl_2_ (Thermo Scientific, Loures, Portugal), 0.2 mM of each dNTP (Fermentas, Loures, Portugal), 0.4 μM of single primer, 1.0 U of *Taq* DNA polymerase (Thermo Scientific, Loures, Portugal) and ultra pure water up to 25 μL. A total of 20 primers (decamer oligonucleotide purchased from Operon Technologies Inc.-OPA) were used to check the fidelity of amplification. Reactions without DNA were used as negative controls. PCR amplification was performed as follows: initial denaturation at 94 °C for 3 min, followed by 30 cycles of 1 min at 94 °C, 1 min at 40 °C and 1 min at 72 °C, and a final extension at 72 °C for 10 min.

ISSR reactions were performed in a volume of 25 μL containing 10 ng of template DNA, 10 × PCR Buffer (10 mM Tris HCl, pH 8.3; 50 mM KCl), 2.5 mM MgCl_2_ (Thermo Scientific, Loures, Portugal), 0.2 mM of each dNTP (Fermentas, Loures, Portugal), 0.4 μM of single primer, 1 U of *Taq* DNA polymerase (Thermo Scientific, Loures, Portugal) and ultra pure water up to 25 μL. A total of 15 primers, designed by Stab Vida (Caparica, Portugal), were screened. Reactions without DNA were used as negative controls. PCR amplification was performed as follows: initial denaturation at 94 °C for 3 min, followed by 35 cycles of 1 min at 94 °C, 1 min at 49 °C, 2 min at 72 °C, and a final 10 min extension at 72 °C.

Amplifications were carried out in a Thermocycler Biometra UNO II (Thermoblock, Biotron). PCR amplifications products were analyzed by electrophoresis at 80 V in 2% (w/v) Wide range/Standard 3:1 Agarose (Sigma, Sintra, Portugal) gels in the presence of a 1 Kb molecular weight marker (Thermo Scientific, Loures, Portugal). Gel was stained with ethidium bromide [27], visualized under a UV transilluminator and photographed using the Stratagene Eagle Eye II. The experiment was repeated twice.

## 4. Conclusions

The Doyle and Doyle [11] protocol was successfully optimized by adding antioxidant compounds to the extraction buffer, by increasing the incubation temperature and by including an extraction with organic solvents. These changes made it possible to obtain high purity DNA from *A. unedo* leaves suitable for further genomic analysis. To our knowledge, no other studies report DNA extraction from this plant. The results obtained will form a strong beginning for future molecular characterization and genetic improvement works in this promising medicinal plant.

## Figures and Tables

**Table 1:** Yield and purity of DNA extracted from *Arbutus unedo* leaves by different methods (mean ± SD; n = 6).

Methods	DNA purity (A_260_/A_280_)	DNA yield (μg/μL)
Doyle and Doyle [11]	n.d.	n.d.
A	1.48 ± 0.448	85.4 ± 23.2
B	1.51 ± 0.304	112.5 ± 48.2
C	1.80 ± 0.021	200.0 ± 78.0

n.d.—Not determined.

## References

[1] Celikel G, Demirsoy L, Demirsoy H (2008). The strawberry tree (*Arbutus unedo* L.) selection in Turkey. Sci. Hortic.

[2] Pedro JG (1994). Carta da Distribuição de Figueira e Medronheiro-Notícia Explicativa.

[3] Alarcão-e-Silva M, Leitão AEB, Azinheira HG, Leitão MCA (2001). The Arbutus Berry: Studies on its color and chemical characteristics at two mature stages. J. Food Compos. Anal.

[4] Mereti M, Grigoriadou K, Nanos GD (2002). Micropropagation of the strawberry tree, *Arbutus unedo* L. Sci. Hortic.

[5] Ziyyat A, Legssyer A, Mekhfi H, Dassouli A, Serhrouchni M, Benjelloun W (1997). Phytotherapy of hypertension and diabetes in oriental Morocco. J. Ethnopharmacol.

[6] Mariotto S, Esposito E, Di Paola R, Ciampa A, Mazzon E, Carcereri de Prati A, Darra E, Vincenzo S, Cucinotta G, Caminiti R (2008). Protective effect of *Arbutus unedo* aqueous extract in carrageenan-induced lung inflammation in mice. Pharmacol. Res.

[7] Afkir S, Nguelefack TB, Aziz M, Zoheir J, Cuisinaud G, Bnouham M, Mekhfi H, Legssyer A, Lahlou S, Ziyyat A (2008). *Arbutus unedo* prevents cardiovascular and morphological alterations in L-NAME-induced hypertensive rats. Part I: Cardiovascular and renal hemodynamic effects of *Arbutus unedo* in L-NAME-induced hypertensive rats. J. Ethnopharmacol.

[8] Oliveira I, Coelho V, Baltasar R, Pereira JA, Baptista P (2009). Scavenging capacity of strawberry tree (*Arbutus unedo* L.) leaves on free radicals. Food Chem. Toxicol.

[9] Bryant JA, Dey PM, Harborne JB (1997). DNA extraction. Methods in Plant Biochemistry.

[10] Zamboni A, Pierantoni L, de Franceschi P (2008). Total RNA extraction from strawberry tree (*Arbutus unedo*) and several other woody-plants. IForest-Biogeosci. For.

[11] Doyle JJ, Doyle JL (1987). A rapid DNA isolation procedure for small quantities of fresh leaf tissue. Phytochem. Bull.

[12] Reichardt M, Rogers S, Ausubel F, Brent R, Kingston RE, Moore DD, Seidman JG, Smith JA, Struhl K (1994). Preparation of plant DNA using CTAB. Current Protocols in Molecular Biology.

[13] Porebski S, Bailey LG, Baum BR (1997). Modification of a CTAB DNA extraction protocol for plants containing high polysaccharide and polyphenol components. Plant Mol. Biol. Rep.

[14] Tel-Zur N, Abbo S, Myslabodski D, Mizrahi Y (1999). Modified CTAB procedure for DNA isolation from epiphytic cacti of the genera *Hylocereus* and *Selenicereus* (*Cactaceae*). Plant Mol. Biol. Rep.

[15] Cheng Y-J, Guo W-W, Yi H-L, Pang X-M, Deng X (2003). An efficient protocol for genomic DNA extraction from *Citrus* species. Plant Mol. Biol. Rep.

[16] Cota-Sánchez JH, Remarchuk K, Ubayasena K (2006). Ready-to-use DNA extracted with a CTAB method adapted for herbarium specimens and mucilaginous plant tissue. Plant Mol. Biol. Rep.

[17] Males Z, Plazibat M, Vundac VB, Zunta I (2006). Qualitative and quantitative analysis of flavonoids of the strawberry tree—*Arbutus unedo* L. (*Ericaceae*). Acta Pharm.

[18] Fiorentino A, Castaldi S, D’Abrosca B, Natale A, Carfora A, Messere A, Monaco P (2007). Polyphenols from the hydroalcoholic extract of *Arbutus unedo* living in a monospecific Mediterranean woodland. Biochem. Syst. Ecol.

[19] Do N, Adams RP (1991). A simple technique for removing plant polysaccharide contaminant from DNA. BioTechniques.

[20] John ME (1992). An efficient method for isolation of RNA and DNA from plants containing polyphenolics. Nucl. Acids Res.

[21] Pirttila AM, Hirsikorpi M, Kamarainen T, Jaakola L, Hohtola A (2001). DNA isolation methods for medicinal aromatic plants. Plant Mol. Biol. Rep.

[22] Peterson DG, Boehm KS, Stack SM (1997). Isolation of milligram quantities of nuclear DNA from tomato (*Lycopersicon esculentum*), a plant containing high levels of polyphenolic compounds. Plant Mol Biol Rep.

[23] Fang GS, Hammar S, Grumet R (1992). A quick and inexpensive method of removing polysaccharides from plant genomic DNA. BioTechniques.

[24] Muhammad AL, Guang-Ning Y, Norman FW, Bruce IR (1994). A simple and efficient method for DNA extraction from grapevine cultivars and *Vitis* species. Plant Mol. Biol. Rep.

[25] Aljanabi SM, Martinez I (1997). Universal and rapid salt-extraction of high quality genomic DNA for PCR-based techniques. Nucl. Acids Res.

[26] Syamkumar S, Lowarence B, Sasikumar B (2003). Isolation and amplification of DNA from rhizomes of turmeric and ginger. Plant Mol. Biol. Rep.

[27] Sambrook J, Fritsh EF, Maniatis T (1989). Molecular Cloning, a Laboratory Manual.

